# Evaluating the Stress Response as a Bioindicator of Sub-Lethal Effects of Crude Oil Exposure in Wild House Sparrows (*Passer domesticus*)

**DOI:** 10.1371/journal.pone.0102106

**Published:** 2014-07-16

**Authors:** Christine R. Lattin, Heather M. Ngai, L. Michael Romero

**Affiliations:** Department of Biology, Tufts University, Medford, Massachusetts, United States of America; University of Tennessee, United States of America

## Abstract

Petroleum can disrupt endocrine function in humans and wildlife, and interacts in particularly complex ways with the hypothalamus-pituitary-adrenal (HPA) axis, responsible for the release of the stress hormones corticosterone and cortisol (hereafter CORT). Ingested petroleum can act in an additive fashion with other stressors to cause increased mortality, but it is not clear exactly why—does petroleum disrupt feedback mechanisms, stress hormone production, or both? This laboratory study aimed to quantify the effects of ingested Gulf of Mexico crude oil on the physiological stress response of house sparrows (*Passer domesticus*). We examined baseline and stress-induced CORT, negative feedback, and adrenal sensitivity in house sparrows given a 1% oil or control diet (n = 12 in each group). We found that four weeks on a 1% oil diet did not alter baseline CORT titers or efficacy of negative feedback, but significantly reduced sparrows' ability to secrete CORT in response to a standardized stressor and adrenocorticotropin hormone injection, suggesting that oil damages the steroid-synthesizing cells of the adrenal. In another group of animals on the same 1% oil (n = 9) or control diets (n = 8), we examined concentrations of eight different blood chemistry parameters, and CORT in feathers grown before and during the feeding experiments as other potential biomarkers of oil exposure. None of the blood chemistry parameters differed between birds on the oil and control diets after two or four weeks of feeding, nor did feather CORT differ between the two groups. Overall, this study suggests that the response of CORT to stressors, but not baseline HPA function, may be a particularly sensitive bioindicator of sub-lethal chronic effects of crude oil exposure.

## Introduction

During disasters such as the 2010 Deepwater Horizon oil spill, as well as during smaller pipeline and tanker spills, animals can be exposed to crude oil both externally and by ingesting oiled food [1]. Surface and subsurface petroleum can persist for decades after a spill [2], [3], and reservoirs of unweathered oil, for example in mangrove sediments, can maintain high toxicity and cause chronic re-contamination of adjacent areas for years [4]. Furthermore, many areas affected by oil spills are used by wildlife as breeding grounds, wintering grounds or sites for migratory stopovers [5]–[7], making it essential to understand the potential physiological impacts of petroleum on wild animals.

Petroleum can disrupt endocrine function in humans and wildlife, with adverse effects on reproductive, thyroid, and stress hormones [8]–[10]. Petroleum interacts in particularly complex ways with the hypothalamus-pituitary-adrenal (HPA) axis, responsible for the release of glucocorticoid hormones (corticosterone and cortisol, hereafter CORT). CORT helps regulate metabolism and the vertebrate stress response, and has a number of other essential roles related to immune function, behavior and reproduction [11]. Petroleum both acts as an environmental stressor and negatively affects an animal's ability to cope with other stressors [12]. This means oil-compromised animals may be less able to cope with even relatively mild stressors, leading to higher mortality than would be predicted from either factor independently. For example, ingested oil decreased the survival of mallard ducks (*Anas platyrhynchos*) subsequently stressed with cold temperatures and saline drinking water [13].

However, despite the importance of the HPA axis in helping animals survive stressors, the effects of oil and other endocrine-disrupting chemicals on the HPA axis are poorly understood [14]. There is some evidence that the adrenal glands may be especially vulnerable to damage from endocrine-disrupting chemicals because of factors such as intense adrenal vascularization and high concentration of enzymes of the cytochrome P450 family, which can cause bioactivation of toxins [15], [16]. But on the most basic level, it is not even clear whether petroleum causes an increase or decrease in plasma concentrations of CORT. In most studies, ingested oil suppresses plasma CORT [8], but some studies report that ingested oil led to elevated plasma CORT, or to no difference [17], [18]. Without a clear understanding of how petroleum affects the HPA axis, it is impossible to know whether animals are successfully coping with the stress of petroleum exposure, or whether they are severely physiologically compromised and hence, less able to deal with additional stressors. A clearer understanding of petroleum's effects on aquatic vertebrates is essential to help us better respond to oil spills and their aftermath (for example, better prioritization of sites to manage), and to fully understand the conservation impacts of these spills.

We sought to clarify petroleum-induced changes to the HPA axis by examining several components of HPA function: baseline and stress-induced CORT titers, negative feedback in response to an injection of dexamethasone (DEX) and adrenal sensitivity in response to an injection of adrenocorticotropin hormone (ACTH). Although relatively few studies use ACTH and DEX injections to look in detail at HPA functioning, these measures provide important insights into HPA regulation [19]–[21]. We conducted these studies in wild-caught house sparrows (*Passer domesticus*) brought into the laboratory. The house sparrow is a common model species for studying avian physiology generally [22] and the HPA axis specifically [21], [23], [24], and it is also easy to catch and keep in captivity. We had three goals in this study: 1) to elucidate what aspects of HPA function are compromised by oil ingestion, 2) to determine if any changes in HPA function could be detected by examining CORT concentrations in bird feathers, and 3) to compare effects on the HPA axis to several other potential biomarkers of oil exposure, including blood chemistry parameters and body weight, to evaluate whether the stress response is a useful bioindicator of sub-lethal chronic effects of crude oil exposure.

## Methods

### Study subjects and experimental diets

For all experiments, wild house sparrows were caught in Medford, MA, USA using seed-baited Potter traps and mist nets at bird feeders. All birds were removed from nets or traps within 15 minutes; no animals were harmed by capture techniques. Upon capture, birds were housed together in an outdoor aviary for up to 2 weeks with *ad libitum* access to mixed seed, grit and water. Once brought into the lab, birds were either individually housed (Pilot study and Experiment 1) or doubly housed (Experiment 2) under natural day length conditions. Sparrows had *ad libitum* access to water, grit and food and all groups fed freely. We did not use gavage or other force-feeding techniques, which could potentially have their own effects on HPA function. Although it is possible that there were differences in food consumption among individuals, there were no differences in body mass between sparrows on the oiled diets and sparrows on control diets (see Results), so birds in the petroleum groups fed to maintain the same body weight as those in the control group. There were equal numbers of males and females in each treatment group, except for Experiment 1, where only males were used because of the need to take testis for another study. Birds were randomly allocated to treatment or control groups.

Oil used was Gulf of Mexico Sweet Louisiana crude obtained from British Petroleum Exploration and Production Inc. (Houston, TX, USA). We weathered oil to ∼75% of original volume by heating at a low temperature and stirring continuously, in order to disperse the most toxic volatile compounds, which do not persist for very long in the environment [25]. Different petroleum doses were mixed into organic sunflower oil (Catania-Spagnia Corporation, Ayer, MA, USA) to create a total volume of 1 mL petroleum and sunflower oil/100 g food, and then mixed with de-husked millet (Agway, Grandin, ND, USA); control birds received de-husked millet and sunflower oil in the same proportions. To compensate for the low diversity of their diet, sparrows in all groups also received Nekton-S multi-vitamin supplement for cage birds (Günter Enderle, Pforzheim, Germany) at a concentration recommended by the manufacturer (0.4 g/100 g of diet).

### Collecting plasma, blood sample processing and radioimmunoassays

For baseline CORT samples, we collected ∼50 µl of blood from each bird from the alar vein within 3 min of a human entering the experimental room. Because CORT titers begin rising ∼2 min after perceiving a stressor in this species (Romero and Reed 2005), all samples collected under 3 min reflect baseline or near baseline levels. Blood was collected in heparinized microhematocrit capillary tubes. To measure stress-induced CORT, birds were restrained in cloth bags for 30 min, after which ∼30 µl of blood was taken. All blood samples were stored on ice, centrifuged at ∼1200 g for 8 min (Centrific Model 225, Fisher Scientific, Pittsburgh, PA, USA), and plasma removed and stored at −20°C.

We determined plasma CORT concentrations using radioimmunoassay (RIA) following the methods of Wingfield et al. [26]. Feather CORT was determined using the RIA method of Bortolotti et al. [27] with some adjustments as in Lattin et al. [28]. We used antibody B3–163 (Esoterix, Calabasas Hills, CA) for plasma RIAs and C 8784 (Sigma-Aldrich, Saint Louis, Missouri, USA) for the feather RIA. For the pilot study RIAs, the average recovery was 84%±1.8% (SEM), and intra- and inter-assay coefficients of variation were 3% and 18%, respectively. For Experiment 1 RIAs, average recovery was 77%±2.4% (SEM), and intra- and inter-assay coefficients of variation were 2% and 10%, respectively. For the feather CORT assay of Experiment 2, all samples were run in the same assay so there was no inter-assay variation; the intra-assay coefficient of variation was 4%.

### Pilot study to determine oil doses

We first conducted a dose-response study using three different concentrations of crude oil mixed into food and a control diet. Most papers report changes in either baseline or stress-induced CORT with oil exposure [18], [29], so we sought to identify the minimum dose causing changes in one or both measures. We also wished to give similar doses to those used in other avian species to facilitate comparison between this and earlier studies. To identify biologically relevant ingestion amounts for crude oil in the environment after a spill, extensive fieldwork would have been necessary, which was beyond the scope of this study.

Wild-caught house sparrows were transferred from an outdoor aviary into the lab and given two weeks to adjust to lab conditions. We tested four treatment groups: no oil, a low oil dose (0.01% oil weight:food weight), a medium oil dose (0.1% weight:weight) and a high oil dose (1% weight:weight) (n = 6 birds in each group). These doses were chosen based on a previous study showing oil ingestion effects on duck plasma CORT at 1.5% and 0.15% doses but not at a 0.015% dose [30].

We took initial blood samples right before starting birds on oiled diets, and then sampled 1x/week for five additional weeks to determine both the degree and timing of changes in baseline or stress-induced CORT. Radioimmunoassays were run on a weekly basis, and we stopped the pilot study at week 5 when we determined an appropriate dose (1% oil weight:food weight - see Results) significantly impacting plasma CORT.

### Experiment 1: The effects of crude oil on the HPA axis of wild-caught house sparrows

This experiment assessed the HPA axis of oil-compromised animals in more depth to answer the following question: is there an easily-identified HPA profile we can use to diagnose oil toxicity in a wild bird? Based on the pilot study (see Results), oil birds (n = 12) received a dose of 1% oil weight:food weight; controls (n = 12) received sunflower oil instead of petroleum. House sparrows were brought into the lab, singly housed and given two weeks to adjust to captivity. Immediately before the onset of feeding, and again two weeks into the feeding experiment, we took blood samples to measure baseline and stress-induced CORT. Four weeks after the onset of feeding, animals were subjected to a set of HPA function tests, which involved taking baseline and stress-induced plasma CORT samples, and then administering injections of two exogenous hormones to better study HPA function.

The ability to suppress CORT output can be assessed using an injection of the synthetic glucocorticoid DEX. DEX binds to CORT receptors and stimulates negative feedback, leading to lower circulating endogenous CORT if feedback is functioning normally [31]–[33]. Immediately after taking stress-induced CORT samples, we gave birds a 1 mg/kg body weight intramuscular injection of DEX (Vedco, St. Joseph, MO). We took a third blood sample of ∼50 µl after an additional 90 min in cloth bags. These are the minimum doses and times required to see a strong feedback effect of DEX in house sparrows, as shown in a previous study [21].

After taking this negative feedback sample, we injected ACTH to stimulate the adrenal glands to release maximal levels of CORT. Sparrows received intramuscular injections of 100 IU/kg body weight porcine ACTH (Sigma Aldrich), a dose shown to be effective in eliciting a maximal response in this species [34]. A fourth and final blood sample of ∼30 µl was collected 15 min later. This resulted in ∼160 ul total blood drawn, below the 1% of body weight per two weeks guideline for birds weighing 27–29 g [35]. We also took initial, two week and four week measurements of body mass using spring scales (Pesola AG, Baar, Switzerland).

### Experiment 2: The effects of ingested crude oil on blood chemistry parameters and feather CORT

In a second experiment using a different group of animals, we measured various blood chemistry parameters. Petroleum ingestion has often been associated with changes in liver function and blood biochemistry [36], [37], which can be assessed using relevant biochemical analytes [38]. Blood chemistry parameters have also been found to track the physiological effects of chronic stress on wild birds [39]. Thus, examining blood chemistry was another way of potentially assessing physiological damage independent of changes in CORT.

We used an Abaxis VetScan Classic analyzer (Model 200–1000, Union City, CA, USA) with Avian/Reptilian Plus Rotors to examine 8 different blood chemistry parameters: aspartate aminotransferase, creatine kinase, uric acid, glucose, phosphorus, calcium ions, total protein and sodium ions, in birds fed oiled (n = 9) and control (n = 8) diets. Abnormal levels of these biochemical analytes can be associated with a range of different pathologies (Table 1). Animals were brought into the lab and control and oil diets prepared as in Experiment 1. Blood chemistry parameters were assessed at two and four weeks after oil feeding. For all VetScan samples, 100 ul of whole blood were drawn from animals within 10 min of entering animal rooms, and kept on ice until they were used in pre-programmed assays within 2 h of collection. These rotors are also supposed to give measures of bile acids, potassium ions, albumin and globulin. However, bile acid concentrations in house sparrow blood were almost always below the dynamic range of the machine (<35 umol/L), and therefore undetectable. Similarly, potassium ions were undetectable in many samples because they were above the machine's dynamic range (>8.5 mmol/L). The VetScan uses the bromcreosol green dye-binding method to assess albumin concentrations, and measured total protein and albumin to calculate globulin concentrations. However, the bromcreosol green dye-binding method is generally considered unreliable in birds [40], [41]. Therefore, these four measures are not included in any analyses.

**Table 1:** List of chemical analytes assessed in whole avian blood using a VetScan machine with Avian/Reptilian Plus rotors, and clinical pathologies these measures can indicate when significantly elevated and/or depressed, according to Harr [35], Fudge [60] and Rosskopf and Woerpel [61].

Parameter	Pathologies indicated
Aspartate aminotransferase	Liver disease; muscle damage
Creatine kinase	Muscle damage
Uric acid	Kidney disease
Glucose	Severe liver disease; sepsis; anorexia; pancreatic disease
Phosphorus	Kidney disease; fluid balance
Calcium ions	Bone disease; kidney disease
Total protein	Liver, gastrointestinal and kidney disease; dehydration; starvation
Sodium ions	Fluid balance; dehydration

In addition, we measured the amount of CORT deposited in feathers grown before and during the feeding experiments. Because we found a significant reduction in stress-induced plasma CORT with oil exposure (see Results), we hypothesized we might also see a reduction in CORT concentrations in feathers grown during the period of oil exposure, which could also potentially serve as a bioindicator of oil exposure. For feather CORT measures, two flight feathers (primary 3 from the left wing and retrix 1 from the left side of the tail) were plucked for initial analysis on 15 May 2013, right before the start of oil feeding, and re-plucked after 5 weeks of feeding, on 19 June 2013, after the feathers had re-grown. In keeping with previous studies, feather CORT was standardized per mm feather [27], [42]. We averaged feather CORT values from the two feather types to determine each individual's pre- and post-feeding experiment sample values. Feathers from one control animal grew much slower and were not full-sized by the end of the study, so were not included in the analysis. Also, one animal from the oil group died during the third week of the study, so its data is only included in biochemical analyses from week 2, not in the biochemical data from week 4 or feather CORT data.

### Data analysis

For the pilot study, we used repeated-measures multivariate analysis of variance (MANOVA) in JMP 10.0 (SAS Institute, Cary, NC, USA) using baseline CORT, stress-induced CORT and body mass obtained across the five weeks of the study as dependent repeated-measure variables and feeding group (0.01%, 0.1% and 1% oil or control) as the independent variable. Specifically, for each measure, when we detected a significant interaction between time and feeding group, we then examined planned contrasts where values from each week of the feeding experiment were compared with initial values (1 week samples compared to initial samples, 2 week samples compared to initial samples, etc.). In cases where these planned contrasts revealed a significant difference, we ran Dunnett's post-hoc tests to determine which oil groups, if any, differed from the control group, as recommended by Ruxton and Beauchamp [43].

To assess negative feedback regulation independently from changes in stress-induced CORT (i.e., compensate for different stress-induced CORT titers used as the starting point for calculating negative feedback), we measured negative feedback as the relative decrease in CORT from stress-induced concentrations: (stress-induced CORT - post-DEX CORT)/(stress-induced CORT) * 100. One hundred percent feedback therefore represents the complete inhibition of CORT from the presumed peak of CORT release after 30 min of restraint.

For Experiment 1, we ran repeated-measures MANOVAs on body mass and baseline and stress-induced CORT using the initial, two week and four week samples as the repeated dependent variables and group (1% oil diet or control) as the independent variable. When we detected a significant overall group effect or an interaction between time and group, we did post-hoc matched t-tests (for overall group effects) or F tests (for group x time interactions). For negative feedback and adrenal sensitivity samples, we only had week 4 samples, so we ran simple t-tests. For Experiment 2, we ran repeated-measures MANOVAs on each of the eight analytes, body mass and feather CORT/mm feather using the two and four week samples as the repeated dependent variables and group (1% oil or control) as the dependent variable. In studies with a balanced design, ANOVA models are fairly robust to violations of normality, but not to violations of homogeneity of variances [44]. Therefore, for each measure, we used Levene's test to test for violations of this assumption [45]. In two cases (creatine kinase and aspartate aminotransferase) we log-transformed data so they would meet this assumption and ran analyses on the transformed data.

### Ethics statement

Animals were monitored daily for changes in weight and behavior due to treatments. All procedures were performed according to Association for Assessment and Accreditation of Laboratory Animal Care (AAALAC) guidelines, and all protocols, including capture techniques, stress protocols, experimental administrations, blood draws, and oil feeding, were approved by the Tufts University Animal Care and Use Committee (protocol #M2012-160).

## Results

### Pilot study

**Figure 1:**
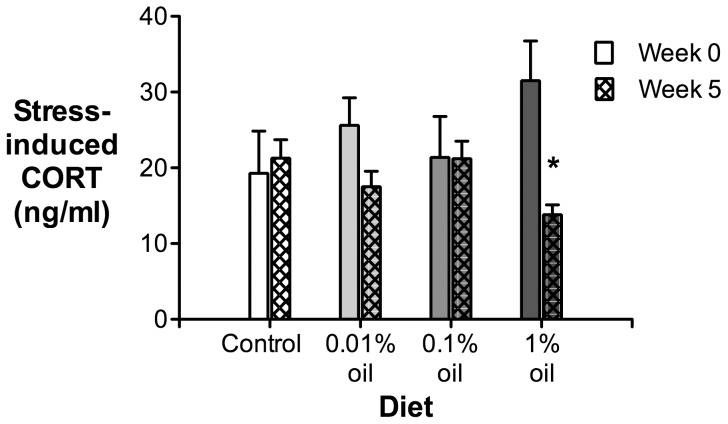
Plasma corticosterone (CORT) titers of house sparrows in response to a standardized stressor (30 min of restraint in a cloth bag) both before (Week 0) and after (Week 5) five weeks of exposure to four different diets: no oil, a low oil dose (0.01% oil weight:food weight), a medium oil dose (0.1% weight:weight) and a high oil dose (1% weight:weight) (n = 6 birds in each group). The star represents a significant decrease in stress-induced CORT titers in the 1% oil dose birds at week 5 compared to controls. Values are presented as mean ± SEM.

After several weeks of feeding the three different oil doses (0.01%, 0.1% and 1% oil:weight food weight), only the 1% dose demonstrated a significant effect on plasma CORT levels compared to controls, and only at Week 5 (Fig 1). Stress-induced CORT was lower in birds on the 1% oil dose compared to controls five weeks after the start of the feeding experiment (full model: F_3,20_ = 3.83, p = 0.0256; Dunnett's post-hoc test for comparison of 1% oil dose birds with controls: p = 0.0168). There were no changes with any dose prior to Week 5 (data not shown). We saw no changes in baseline CORT or body mass among any of the groups compared to controls (data not shown).

### Experiment 1

**Figure 2:**
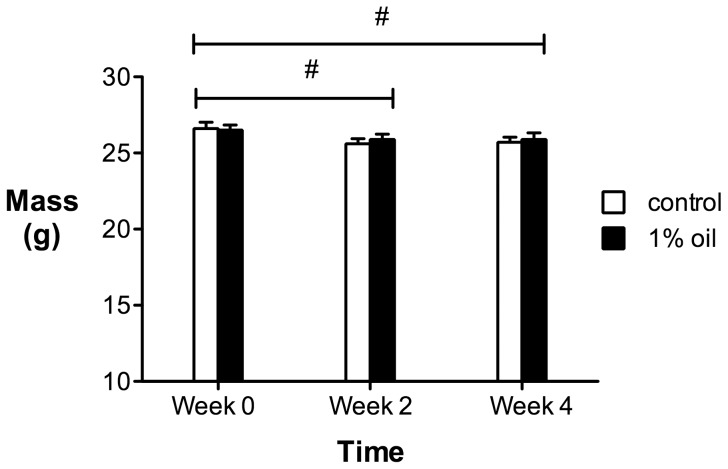
Body mass of house sparrows over four weeks of exposure to a 1% oil or control diet (n = 12 birds in each group). The hash represents a small but significant decrease in body mass of both groups at two and four weeks compared to at the initiation of the feeding study. Values are presented as mean ± SEM.

Body mass did not differ between birds on a 1% oil diet and controls (Fig 2; Treatment effect: F_1,22_ = 0.049, p = 0.83), although there was a significant effect of time on body mass that was the same for both groups (Time effect: F_2,21_ = 13.01, p = 0.0002; Time x treatment interaction: F_2,21_ = 0.97, p = 0.40). Body mass in both groups was higher before the onset of the feeding experiment than it was during week 2 (t = −4.98, df = 23, p = 0.0001) or week 4 (t = −4.50, df = 23, p = 0.0002); there was no difference between week 2 and week 4 body mass (t = 0.18, df = 23, p = 0.86).

**Figure 3:**
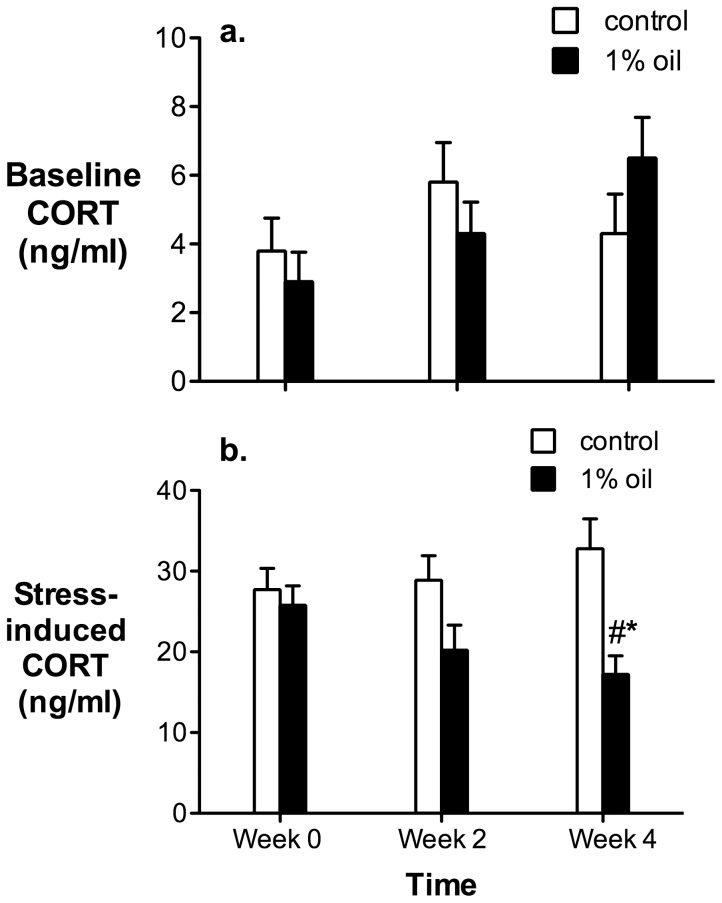
Baseline (a) and stress-induced (b) corticosterone (CORT) titers of house sparrows before initiation of a feeding study (Week 0), and then two weeks and four weeks after exposure to a 1% oil or a control diet (Week 2 and Week 4) (n = 12 birds in each group). The hash represents a significant decrease in stress-induced CORT titers in the birds on the 1% oil diet at four weeks compared to their own values at the initiation of the feeding study; the star represents a significant decrease in stress-induced CORT titers in the birds on the 1% oil diet compared to the controls at 4 weeks. Values are presented as mean ± SEM.

There were no differences in baseline CORT between birds on a control diet and birds on a 1% oil diet (Fig 3a; Treatment effect: F_1,22_ = 0.0056, p = 0.94; Time effect: F_2,21_ = 2.76, p = 0.086; Time x treatment interaction: F_2,21_ = 1.96, p = 0.17). However, there was an overall significant effect of diet on stress-induced CORT (Fig 3b; Treatment effect: F_1,22_ = 7.06, p = 0.014), as well as a significant time x treatment interaction (Time effect: F_2,21_ = 0.59, p = 0.56; Time x treatment interaction: F_2,21_ = 5.28, p = 0.014). Post-hoc analysis of this interaction effect revealed that birds on a 1% oil diet had significantly lower stress-induced CORT at week 4 compared to their initial samples (F_1,22_ = 11.06, p = 0.0031).

**Figure 4:**
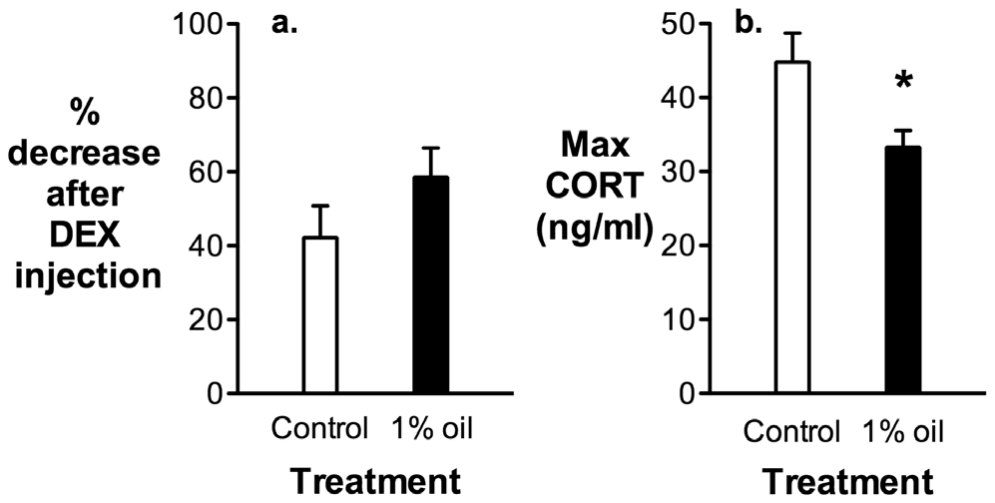
Negative feedback in response to an injection of dexamethasone (DEX) (a) and maximum corticosterone (CORT) secretion in response to an injection of adrenocorticotropin hormone (b) in house sparrows after four weeks of exposure to a 1% oil or a control diet (n = 12 birds in each group). The star represents a significant decrease in adrenal sensitivity in birds on the oil diet compared to controls. Values are presented as mean ± SEM.

After 4 weeks of the feeding experiment, there was no difference in negative feedback regulation between birds on a 1% oil diet and controls (Fig 4a; t = 1.95, df = 22, p = 0.18). However, the ability to secrete CORT after an injection of ACTH was significantly reduced in birds on the 1% oil diet (Fig 4b; t = 6.40, df = 22, p = 0.019).

### Experiment 2

**Figure 5:**
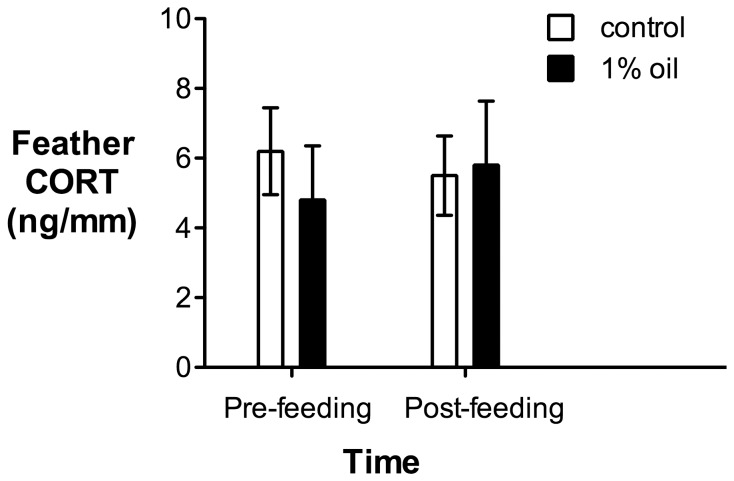
Corticosterone (CORT) concentrations in house sparrow feathers plucked before (pre-feeding) and after (post-feeding) five weeks of exposure to either a 1% oil (n = 8) or a control diet (n = 7). Each animal's feather CORT value was determined by averaging feather CORT concentrations from two different flight feathers - primary 3 from the left wing and retrix 1 from the left side of the tail. Values are presented as mean ± SEM.

Once again, overall body mass did not differ between animals on a control or a 1% oil diet (Table 2; Treatment effect: F_1,14_ = 0.070, p = 0.80) and there were no changes from week 2 to week 4 (Time effect: F_1,14_ = 3.13, p = 0.10; Time x treatment interaction: F_1,14_ = 0.65, p = 0.44). Similarly, there were no differences in aspartate aminotransferase, uric acid, glucose, phosphorus, calcium ions, total protein or sodium ions between animals on a control or a 1% oil diet (Table 3). We did see a significant time effect on creatine kinase concentrations (Table 3), but no difference between animals fed a 1% oil or control diet and no interaction, indicating that this effect was the same for both groups. Feather CORT also did not vary between feathers of animals fed a control or a 1% oil diet (Fig 5; Treatment effect: F_1,13_ = 0.094, p = 0.76; Time effect: F_1,13_ = 0.043, p = 0.84; Time x treatment interaction: F_1,13_ = 1.04, p = 0.33).

**Table 2:** House sparrow whole blood chemistry values in animals fed an oiled diet (1% oil weight:food weight; n = 9) or a control diet (n = 8).

	Oiled diet		Control diet	
Parameter (units)	Week 2	Week 4	Week 2	Week 4
Body mass (g)	24.9±2.0 (22–28.5)	25.1±2.2 (22–28)	24.9±1.1 (23–26)	25.4±1.0 (24–27)
Aspartate aminotransferase (IU/L)	528.7±482.8 (252–1749)	329.1±67.7 (261–439)	408.7±321.0 (236–1133)	296.0±32.6 (239–336)
Creatine kinase (IU/L)	573.8±275.5 (226–1017)	505.0±155.6 (313–749)	1070.4±1398.3 (329–4452)	382.7±139.0 (210–601)
Uric acid (mg/dL)	14.1±4.2 (5–1)	14.5±3.7 (9–20)	17.0±4.4 (10–25)	14.3±3.2 (10–18)
Glucose (mg/dL)	382.0±28.2 (348–436)	377.8±34.7 (319–431)	383.9±95.6 (296–600)	369.4±39.7 (316–440)
Phosphorus (mg/dL)	5.3±1.5 (1.9–6.6)	5.6±1.4 (3.6–7.5)	5.2±1.7 (3.4–8.6)	4.2±0.4 (3.5–4.6)
Calcium ions (mg/dL)	8.3±0.5 (7. 5–9. 1)	8.4±0.5 (7.6–9.3)	8.5±0.6 (7.7–9.7)	8.1±0.5 (7.5–9.0)
Total protein (g/dL)	3.4±0.5 (2.9–4.2)	3.4±0.3 (2.8–3.8)	3.3±0.3 (2.7–3.6)	3.3±0.4 (2.7–3.8)
Sodium ions (mmol/L)	158.6±3.7 (153–163)	159.3±3.0 (154–163)	158.6±8.5 (141–168)	155.1±2.4 (152–159)

In some cases, blood volume was not sufficient for all analyses, and one animal from the oil group died during week 3 (oiled birds: n = 8 for creatine kinase during week 2, and n = 8 for all analyses during week 4; control birds: n = 7 for aspartate aminotransferase during week 2, and n = 7 for all analyses during week 4). Values were determined using Avian/Reptilian Plus rotors on a portable VetScan machine. Values are presented as mean ± SD (range).

**Table 3:** Statistical analyses of house sparrow whole blood chemistry values in animals fed an oiled diet (1% oil weight:food weight) or a control diet.

	Oiled diet vs control diet		
Parameter	df	F	p
Aspartate aminotransferase			
*treatment*	1, 12	0.50	0.49
*time*	1, 12	1.97	0.19
*time x treatment*	1, 12	0.014	0.91
Creatine kinase			
*treatment*	1, 12	0.0006	0.98
*time*	1, 12	5.32	0.040*
*time x treatment*	1, 12	1.68	0.22
Uric acid			
*treatment*	1, 13	0.94	0.35
*time*	1, 13	0.66	0.43
*time x treatment*	1, 13	2.15	0.17
Glucose			
*treatment*	1, 13	0.019	0.89
*time*	1, 13	0.32	0.58
*time x treatment*	1, 13	0.077	0.79
Phosphorus			
*treatment*	1, 13	1.85	0.20
*time*	1, 13	0.26	0.62
*time x treatment*	1, 13	1.80	0.20
Calcium ions			
*treatment*	1, 13	0.10	0.76
*time*	1, 13	0.86	0.37
*time x treatment*	1, 13	1.51	0.24
Total protein			
*treatment*	1, 13	0.23	0.64
*time*	1, 13	0.12	0.73
*time x treatment*	1, 13	0.12	0.73
Sodium ions			
*treatment*	1, 13	1.30	0.28
*time*	1, 13	0.31	0.59
*time x treatment*	1, 13	1.11	0.31

*Significant at the p<0.05 level. See Methods for details on statistical procedures, and Table 2 caption for sample sizes. Values were determined using Avian/Reptilian Plus rotors on a portable VetScan machine.

## Discussion

Compared to other physiological measures we examined, including eight different blood chemistry parameters, certain components of the HPA axis were more sensitive to disruption by the toxins in weathered crude oil. Specifically, birds fed a 1% oil diet for four weeks secreted less CORT in response to a standardized stressor and an ACTH injection compared to control animals. Although we also saw a slight but significant drop in body mass at two and four weeks compared to initial values, this decrease was the same in both groups of animals, indicating that it was caused by the switch from a diverse, mixed-seed diet to a diet consisting solely of millet and a vitamin supplement. Similarly, there was an overall significant effect of time on creatine kinase levels. Because this measure can reflect muscle damage, this increase could be caused by repeated capture and restraint for blood sampling [46].

Our ACTH injection data suggest that reductions in stress-induced CORT in animals exposed to an oil diet may be due to changes in the CORT-secreting cells of the adrenal gland. Although a previous study found no difference in adrenal mass in mallard ducks ingesting an oil diet compared to controls, slices of adrenal tissue from oil-fed ducks secreted less CORT when cultured with ACTH [47]. There is ample evidence that the steroid hormone biosynthesis pathway can be disrupted by endocrine-disrupting chemicals [15], [48], suggesting this as the specific mechanism altering CORT release in our study animals. Because reductions in stress-induced CORT titers after ingesting oil have now been seen in mallard ducks [47] and sparrows (this study), two distantly related avian species, these findings seem to be generalizable across a wide range of birds. These results also suggest that disrupted HPA function may be a particularly useful sub-lethal indicator of exposure to crude oil, even more than some physiological measures that are often used, such as plasma concentrations of aspartate aminotransferase [49]. However, it should be emphasized that our dosing regimen used chronic exposure to a relatively low dose of petroleum, and other physiological measures could be better indicators of exposure to a short-term but higher dose of petroleum.

The technique of plucking bird feathers to sample for hormones is a relatively new one, but has been enthusiastically embraced by avian ecologists and conservationists because it can potentially provide an integrated measure of a bird's circulating hormones during the period of feather growth [27], [50]. Because stress-induced plasma CORT was significantly reduced in animals fed an oiled diet, we hypothesized we might also see a reduction in CORT concentrations in feathers grown during oil exposure. However, contrary to our hypothesis, there was no difference in feather CORT in animals fed an oiled or a control diet. It takes several weeks to complete feather growth. During most of this period, plasma CORT will be at normal baseline concentrations, with only occasional elevations due to stressor exposure [51]. We saw no difference in baseline CORT titers between birds on an oiled diet compared to controls, so feather CORT may reflect baseline CORT more than stress-induced CORT. In any case, these data demonstrate that feather CORT does not reflect the reduction in stress-induced CORT that occurs in animals that have ingested oil.

This study adds to a growing body of work showing that the HPA axis is sensitive to endocrine disruption by a wide variety of different toxicants, including mercury, heavy metals, PCBs, and dioxins [52]–[56]. This is important because animals that are unable to mount a glucocorticoid response are more likely to die when exposed to additional stressors [13], [57], [58], which means that endocrine disruption of the HPA axis can negatively impact the fitness of wild animals. Researchers trying to determine whether animals have been exposed to toxicants should consider assessing stress-induced CORT titers both in response to a standardized stressor as well as in response to an injection of ACTH, the two measures that showed significant changes in our study animals even at fairly low samples sizes. Although in many animals stress-induced CORT can vary naturally at different life history stages [59], in a previous study we found that adrenal sensitivity was generally invariant in wild house sparrows from one life history stage to the next, with the exception of a drop in adrenal sensitivity immediately prior to and during molt [21]. Therefore, adrenal sensitivity may be a particularly useful measure, although appropriate ACTH doses to elicit maximal adrenal output would have to be validated in any new species. Future studies should determine how long these effects persist after oil exposure ends, and assess whether animals can compensate for decreased circulating CORT by increasing tissue sensitivity to hormone (i.e., by increasing tissue concentrations of CORT receptors).

## Supporting Information

Table S1 **Pilot experiment data file.**
(XLSX)

Table S2 **Experiment 1 data file.**
(XLSX)

Table S3 **Experiment 2 data file.**
(XLSX)
